# Dietary Supplementation of Compound Probiotics Improves Intestinal Health by Modulated Microbiota and Its SCFA Products as Alternatives to In-Feed Antibiotics

**DOI:** 10.1007/s12602-024-10314-3

**Published:** 2024-06-21

**Authors:** Wenxing Wang, Guoqi Dang, Wei Hao, Anping Li, Hongfu Zhang, Shu Guan, Teng Ma

**Affiliations:** 1 State Key Laboratory of Livestock and Poultry Nutrition and Feeding, Institute of Animal Sciences, Chinese Academy of Agricultural Sciences, Beijing, 100193 China; 2 Department of Animal Nutrition and Health, DSM (China) Co., Ltd, Shanghai, 201203 China; 3 Department of Animal Nutrition and Health, DSM Singapore Industrial Pte. Ltd, Singapore, 117440 Singapore

**Keywords:** Compound probiotics, Digestive enzymes, Microbiota, SCFAs, Intestinal morphology

## Abstract

*Enterococcus faecium*, *Bifidobacterium*, and *Pediococcus acidilactici*, as intestinal probiotics, have been proved to play a positive role in treating intestinal diseases, promoting growth and immune regulation in poultry. The aim of this study was to evaluate the effect of compound probiotics on growth performance, digestive enzyme activity, intestinal microbiome characteristics, as well as intestinal morphology in broiler chickens. Treatment diets with chlortetracycline and compound probiotics were used for two groups of sixty broilers each throughout the feeding process. Another group was fed the basal diet. The BW (2589.41 ± 13.10 g vs 2422.50 ± 19.08 g) and ADG (60.57 ± 0.31 g vs 56.60 ± 0.45 g) of the compound probiotics added feed treatment group were significantly increased, and the FCR was significantly decreased (*P* < 0.05). The supplementation of a compound probiotics enhanced the abundance of beneficial bacteria such as *Lactobacillus*, *Faecalibacterium*, and *norank_f_norank_o_Clostridia_vadinBB60_group* (*P* < 0.05), and modulated the cecal microbiota structure, thereby promoting the production of short-chain fatty acids (SCFAs) and elevating their levels (*P* < 0.05), particularly propionic and butyric acids. Furthermore, the administration of the compound probiotics supplements significantly enhanced the villi height, V/C ratio, and reduced the crypt depth (*P* < 0.05). In addition, the activity of digestive enzymes in the duodenum and jejunum was elevated (*P* < 0.05). Collectively, the selected compound probiotics supplemented in this experiment have demonstrated efficacy, warranting further application in practical production settings as a viable alternative to antibiotics, thereby facilitating efficient production and promoting gastrointestinal health.

## Introduction

Over the years, chicken production increased, making great contributions to human protein supply and becoming an indispensable source of animal protein [1]. The high-quality and efficiency broiler products is inseparable from the health of animals in the process of farming. Antibiotics have been used in the poultry industry for decades to combat infectious pathologies and perform preventative treatment. As additives in animal feed, antibiotics act excellently on improving feed efficiency and production performance [2–4]. For example, chlortetracycline is a typical antibiotic capable of fighting Gram-positive and Gram-negative bacteria, as well as some protozoa and mycobacteria. It is commonly used as a feed additive to promote growth and provide a good disease control [5].

Antibiotic drugs are commonly used in poultry for three purposes: (1) therapeutic use, where animals (individual or small groups) are used of high doses of antibiotics over a relatively short period of time; (2) prophylactic use, in which animals are exposed to moderate doses of antibiotics for longer time duration; and (3) growth promoting use, that is, subtherapeutic doses of antibiotics promote growth [4]. However, excessive and blind misuse of antibiotics in production and treatment for the purpose of growth promotion has accelerated the development of antibiotic resistance and the accumulation of residues in animal products.. Moreover, antibiotic use disrupts the intestinal microbiome and intestinal barrier, resulting in impaired intestinal immune function and threatening the health of animals themselves [6, 7]. In the process of the use of antibiotics in broiler breeding, only a small part of antibiotics can be utilized by the body to produce corresponding effects, while most of the rest antibiotics may accumulate in the body of broiler and induce antibiotic resistance genes with microbial flora [8, 9]. Therefore, producing broilers without antibiotics is critical to saving poultry and public health in the fight against antimicrobial resistance. In recent years, the use of feed additives to replace the addition of antibiotics has become a hot topic, including probiotics, prebiotics, organic acids, exogenous enzymes, and plant extracts [10, 11].

Increasing evidence has shown that intestinal microbiota play important role in keeping host health, suggesting the potential of manipulation of intestinal microbiota through probiotics practicing to maintain animal health [12]. Probiotics and antimicrobial peptides (AMPs) are two promising approaches that have shown potential benefits in various diseases. Probiotics are live microorganisms that confer health benefits to the host when administered in adequate amounts [13]. AMPs, usually produced with probiotic bacteria, are short amino acid sequences that have broad-spectrum activity against bacteria, fungi, viruses, and parasites. Both probiotics and AMPs can modulate the host immune system, inhibit the growth and adhesion of pathogens, disrupt biofilms, and enhance intestinal barrier function. Some studies have shown that intestinal symbiotic bacteria could induce AMPs and the presence of antibiotics such as penicillin could decrease the expression of AMPs genes [14]. The use of AMPs is more inclined to directly inhibit the growth of harmful bacteria and has a strong bactericidal effect. One of the advantages of using AMPs is that microorganisms cannot develop a resistance to them; therefore, they may represent an important tool in the treatment of MDR bacterial infections [15]. Unfortunately, there are some drawbacks for the immediate use of natural AMPs in clinical practice as they are susceptible to proteolytic degradation and have low oral bioavailability [16]. Therefore, in broiler production, probiotics are more universal, can provide multiple benefits, and play an important role in regulating intestinal microbial communities, promoting nutrient absorption and enhancing immunity.

*Enterococcus faecium*, as a lactic acid bacterium (LAB), can effectively improve the growth performance of animals, reduce mortality, improve intestinal morphology, beneficially regulate the intestinal microbiome of broilers, and can improve Phosphorus absorption and bone mineralization, promote healthy growth of animals, and prevent the occurrence of diseases [17, 18]. In the same way, *Bifidobacterium* is used as a substitute for growth-promoting antibiotics, which can effectively improve the body weight and feed conversion rate of broilers, thereby improving the growth performance of broilers [19]. It has been found that the combination of oligosaccharides and *Bifidobacterium*, as a superior product to replace antibiotics in broiler production, can not only improve growth efficiency, but also establish a favorable intestinal microbiome composition, thereby giving the host intestinal health benefits [20]. *Pediococcus acidilactici*, one of the probiotics commonly used in broiler production, showed an important advantage in the production process is that it can balance the ratio of *Firmicutes* to *Bacteroidota* to modulate intestinal microbiome homeostasis and reduce the abundance of pathogenic enterobacteria [21, 22].

From what has been discussed above, these studies strongly showed that adding probiotics to the diet can improve nutrient utilization and promote growth. Nevertheless, less research has focused on the role of compound probiotics in broiler production. Therefore, the purpose of this experiment was to investigate the effects of compound probiotics additives composed of *Enterococcus faecium*, *Bifidobacterium* and *Pediococcus acidilactici* on growth performance, digestive enzyme activity, short-chain fatty acids, intestinal flora, and intestinal characteristics of broiler chickens.

## Materials and Methods

### Compound Probiotics and Antibiotic

The compound probiotics supplement (DSM Singapore Industrial Pte. Ltd.) was composed of three strains of *Enterococcus faecium*, *Bifidobacterium*, and *Pediococcus acidilactici*, by using oligosaccharides and inulin as carriers. The compound probiotics supplemented to diet at a level of 1000 mg/kg, in which the strain dose of *Enterococcus faecium*, *Bifidobacterium*, and *Pediococcus acidilactici* was 2 × 10^10^ CFU/g, 1 × 10^10^ CFU/g, and 3 × 10^9^ CFU/g, respectively. The addition amount of chlortetracycline (Wuhan Ammunition Life-tech Co., Ltd., Wuhan, China) was 80 mg/kg.

### Broiler, Diet, and Housing Management

A total of 180 1-day-old Arbor Acres (AA) broilers were weighted and divided into three groups as control group (CON), chlortetracycline antibiotics group (CTC), and compound probiotics supplementation group (PSM) in a completely randomized design. There were ten broilers assigned in a cage, and six replicates of cages in each treatment. The corn-based basal diets were formulated for grower from day 1 to 21 and finisher from day 22 to 42 (Table 1). All diets met or exceeded nutrient requirements of broilers according to the provisions of National Research Council (NRC, 1994). Feed and water were supplied ad libitum.

**Table 1:** Ingredients and chemical composition of the basal diets

Items	Grower diet (D1–D21)	Finisher diet (D22–D42)
Ingredient, %		
Corn (CP 7.8%)	57.00	61.00
Soybean meal (CP 43.5%)	35.80	31.80
Soybean oil	1.40	2.90
Wheat middings	1.67	0.97
Limestone	1.28	1.01
Salt	0.35	0.35
Monocalcium phosphate	1.75	1.39
Choline chloride (50%)	0.26	0.20
DL-methionine (98%)	0.20	0.12
L-lysine (99%)	0.05	0.01
Premix^a^	0.04	0.05
Premix^b^	0.20	0.20
Total	100	100
Nutritional ingredient		
ME (MJ/kg)	2.95	3.05
CP (%)	21.00	19.50
Ca (%)	1.00	0.80
Available phosphorus (%)	0.45	0.38
Lysine (%)	1.20	1.05
Methionine (%)	0.52	0.42

^a^Each kilogram of diet provides: vitamin A, 9140 IU; dimension D3, 4,405 IU; E, 11 IU; water-soluble vitamin K, 7.30 mg; riboflavin, 9.15 mg; D-pantothenic acid, 18.33 mg; nicotinic acid, 73.50 mg; choline chloride, 1285 mg; B12, 200 µg; biotin, 900 µg; amine nitrate, 3.67 mg; folic acid, 1650 µg; pyridoxine acid, 5.50 mg

^b^I, 1.85 mg; Mn, 110.10 mg; Cu, 7.40 mg; Fe, 73.50 mg; Zn, 73.50 mg; Se, 500 µg

Broilers were kept in three-layer cages with length, width and height of 150 cm × 60 cm × 70 cm for one cell. The initial room temperature was 33 ℃ in the first 3 days and gradually decreased to 24 ℃ at 28 days of age. After that, the temperature was kept at 22 ~ 24 ℃ until the end of the experiment. The light schedule was a cycle of 23-h light and 1-h darkness throughout the entire experiment.

### Growth Performance and Sample Collection

Broiler body weight and their feed in each replicate cage were evaluated weekly (D1, D7, D14, D21, D28, D35, and D42). The average body weight (ABW), average daily gain (ADG), and average daily feed intake (ADFI) of each treatment were calculated during the grower (D1–D21), finisher (D21–D42), and overall periods (D1–D42). The ratio between ADFI and ADG (F/G) was also calculated from ADFI and ADG data.

At D21 and D42, one chicken with the body weight close to the average in each cage was euthanized for tissue sample collection. There were six individuals from each treatment. The intestinal tissue of duodenum and jejunum was stored at polyformaldehyde to test intestinal histomorphological analysis, and intestinal contents of duodenum, jejunum, and cecum were collected and stored at −80 ℃ to test digestive enzyme activity, SCFA concentration, and intestinal microbiome.

### Digestive Enzyme Activity in Duodenum and Jejunum

The activities of amylase, lipase, trypsin, and chymotrypsin in the intestinal contents were measured with commercial kits (Nanjing Jian Cheng Bioengineering Institute, Nanjing, China) following the instructions of the manufacturer. To quantify digestive enzyme activity, 100 mg of each intestinal content was removed into a centrifugal tube and the supernatant was harvested after centrifugation at 4000 rpm/min for 10 min at 4 ℃. The obtained supernatant is performed according to the instructions of the digestive enzyme kit to be measured.

### SCFA Concentration in Cecum

The short-chain fat acids (SCFA, including acetic acid, propanoic acid, isobutyric acid, butyric acid, isovaleric acid and valeric acid) in cecal digest were determined by gas chromatography (GC) method according to the described of Dang et al. [23]. SCFAs in the digest were extracted by ultrapure water at 10,000 × g centrifugation, and 25% metaphosphoric acid was mixed with the extracts at a ratio of 1:9. After centrifugation of 12,000 × g, the mixture was passed through the 0.45-µm Milled-LG filter (Millipore, Billerica, MA, USA) and subjected for SCFA analysis with the Agilent 7890 N gas chromatograph (Agilent, Santa Clara, CA, USA).

### Intestinal Histomorphological Analysis

Hematoxylin and eosin (H&E) staining was used to analyze intestinal histomorphology including villus height, crypt depth, and V/C ratio cited as described by Yin et al. [24]. The duodenum and jejunum samples were dehydrated, embedded in paraffin, and then sliced at 5 µm thickness. The samples were stained with H&E and observed on a Leica DM2000 light microscope (Leica Microsystems, Wetzlar, Germany). The images were analyzed with ImageJ version 1.8 software (National Institutes of Health, MD, USA). Two replicates of complete villus and crypt from each histological section were selected for measurement.

### DNA Extraction, Amplification, and Sequencing

According to the manufacturer’s instructions, the bacterial DNA of cecal digest samples was extracted with a PowerSoil DNA Isolation Kit (MoBio Laboratories, Carlsbad, CA, USA). The V3-V4 regions of the 16S rRNA gene were amplified by an ABI GeneAmp® 9700 PCR thermocycler (ABI, Foster, CA, USA). The primers were 338F (5ʹ-ACTCCTACGGGAGGCAGCAG-3ʹ) and 806R (5ʹ-GGACTACHVGGGTWTCTAAT-3ʹ). PCR amplification reactions were triplicated and then purified by AxyPrep DNA Gel Extraction Kit (Axygen Bio-sciences, Union City, CA, USA). Purified amplicons were pooled in equimolar and paired end sequenced on an Illumina MiSeq platform (Illumina, San Diego, USA) according to the standard protocols by Majorbio Bio-Pharm Technology Co. Ltd. (Shanghai, China), as previously described [25]. The raw reads were deposited into the NCBI Sequence Read Archive (SRA) database.

Raw fastq files were demultiplexed, quality-filtered by QIIME (version 1.70). Operational taxonomic units (OTUs) were clustered using a 97% similarity cutoff with UPARSE (version 7.1), and chimeric sequences were removed using UCHIME (version 7.1). OUT representative sequence was obtained based on RDP classifier [26].

### Statistical Analysis

The statistical analyses were performed using SPSS statistical program (IBM, version 20, Chicago, IL, USA, 2011). The data were analyzed using the general linear model (GLM) procedure. Analysis of variance (ANOVA) tests were used for analyses of variance accompanied by Duncan’s multiple range test to detect the differences between the treatments. Moreover, ANOVA with repeated measurements was applied for BW, AD, feed intake, and FCR results. The results are presented as mean ± standard error of the mean. Probability values less than 0.05 (*P* < 0.05) were considered significant.

## Results

### Growth Performance

The effects of compound probiotics supplementation on growth performance of broilers are shown in Table 2. In all groups, there was no discernible variation in the initial body weight of broilers. Broilers fed with the diet of chlortetracycline and compound probiotics showed better growth performance compared to the CON group, which was characterized by the significant increase in BW and ADG, reduced FCR at phase 1 and phase 2, and all growing cycle (*P* < 0.05) and feed intake were not affected by supplementation (*P* > 0.05). For all phases, PSM group had no significant differences between CTC group on BW, ADG feed intake, and FCR. The surviving rates were 100% in all treatments. Altogether, these findings demonstrated that compound probiotics had effective effects on the growth performance of broilers.

**Table 2:** Growth performance of the broilers in different supplementation groups (mean ± SEM)

Items	CON	CTC	PSM	*P*-value
Initial weight, g	45.50 ± 0.20	45.35 ± 0.10	45.50 ± 0.18	0.784
D1–D21 (phase 1)				
BW^A^, g	617.27 ± 6.34^b^	649.67 ± 7.32^a^	648.22 ± 8.31^a^	0.011
ADG^B^, g	27.23 ± 0.30^b^	28.78 ± 0.35^a^	28.70 ± 0.40^a^	0.011
Feed intake, g	893.32 ± 14.75	888.28 ± 7.91	887.00 ± 6.84	0.903
FCR^C^	1.56 ± 0.02^a^	1.47 ± 0.02^b^	1.47 ± 0.02^b^	0.010
D22–D42 (phase 2)				
ADG, g	85.96 ± 0.76^b^	93.49 ± 3.03^a^	92.44 ± 0.58^a^	0.023
Feed intake, g	2660.63 ± 20.83	2608.55 ± 53.85	2660.47 ± 25.64	0.523
FCR	1.44 ± 0.01^a^	1.30 ± 0.03^b^	1.34 ± 0.02^b^	0.001
D1–D42				
BW, g	2422.50 ± 19.08^b^	2612.91 ± 63.78^a^	2589.41 ± 13.10^a^	0.007
ADG, g	56.60 ± 0.45^b^	61.13 ± 1.52^a^	60.57 ± 0.31^a^	0.007
Feed intake, g	3553.94 ± 23.99	3496.83 ± 59.47	3547.47 ± 28.94	0.565
FCR	1.50 ± 0.01^a^	1.36 ± 0.02^b^	1.39 ± 0.01^b^	< 0.001
Survival rate, %	100	100	100	1

^a,b^Means within the same row (in each trial independently) with different superscripts are significantly different (*P* < 0.05).

^A^BW means body weight, weighing at D21 and D42

^B^ADG = (Final body weight − Initial body weight)/Feeding time (days)

^C^FCR = Feed intake (in grams)/weight gain (in grams)

### Digestive Enzymes Activity in Duodenum and Jejunum

**Fig. 1:**
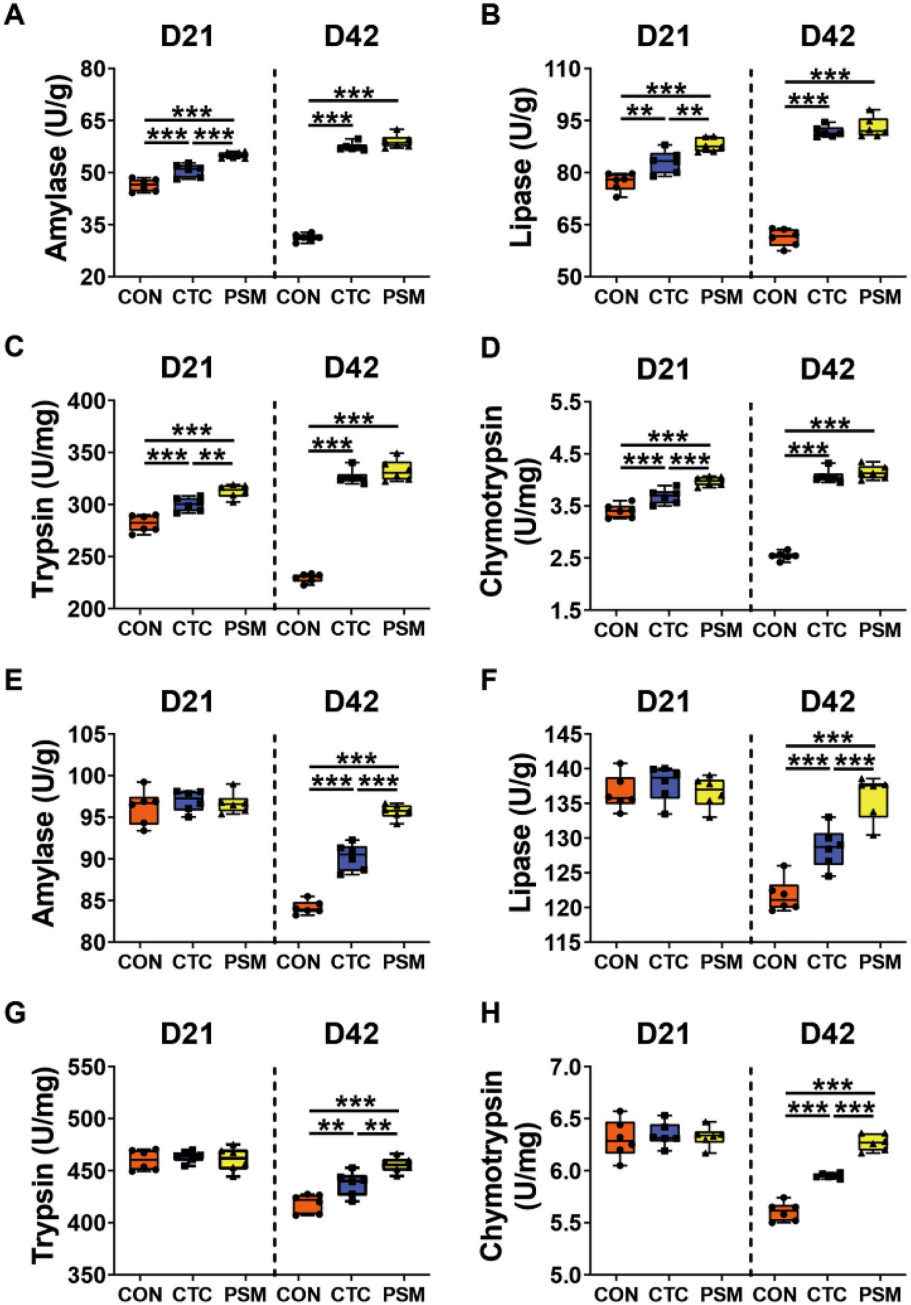
Effects of compound probiotics on digestive enzyme activity in duodenum and jejunum of broilers. **A** Amylase in duodenum; **B** lipase in duodenum; **C** trypsin in duodenum; **D** chymotrypsin in duodenum; **E** amylase in jejunum; **F** lipase in jejunum; **G** trypsin in jejunum; **H** chymotrypsin in jejunum (*n* = 6/group). Data is presented as the mean ± SEM. Signification is presented as *P < 0.05, **P < 0.01, and ***P < 0.001

The activities of digestive enzymes (amylase, lipase, trypsin and chymotrypsin) of broilers intestinal contents are presented in Fig. 1. According to the findings, CTC group and PSM group significantly increased the activity of digestive enzymes in the duodenum compared to the CON group (*P* < 0.01; Fig. 1A–D). Furthermore, at D21, the activity of amylase, lipase, trypsin, and chymotrypsin in PSM group was considerably higher than that in CTC group (*P* < 0.01). However, there was no distinction in the activity of these four digestive enzymes between PSM group and CTC group at D42 (*P* > 0.05). For jejunum (Fig. 1E–H), at D21, there was no significant difference in the digestive enzyme activities between CON group, CTC group, and PSM group (*P* > 0.05), whereas, at D42, digestive enzyme activities in PSM group were the highest, followed by these in CTC group, which were noticeably higher than these in CON group, and reached significant level (*P* < 0.01). These results indicated that compound probiotics was effective in improving the intestinal digestive enzyme activity of broilers, and the effect was better than chlortetracycline.

### Composition and Variation of Microbiota in Cecum

**Fig. 2:**
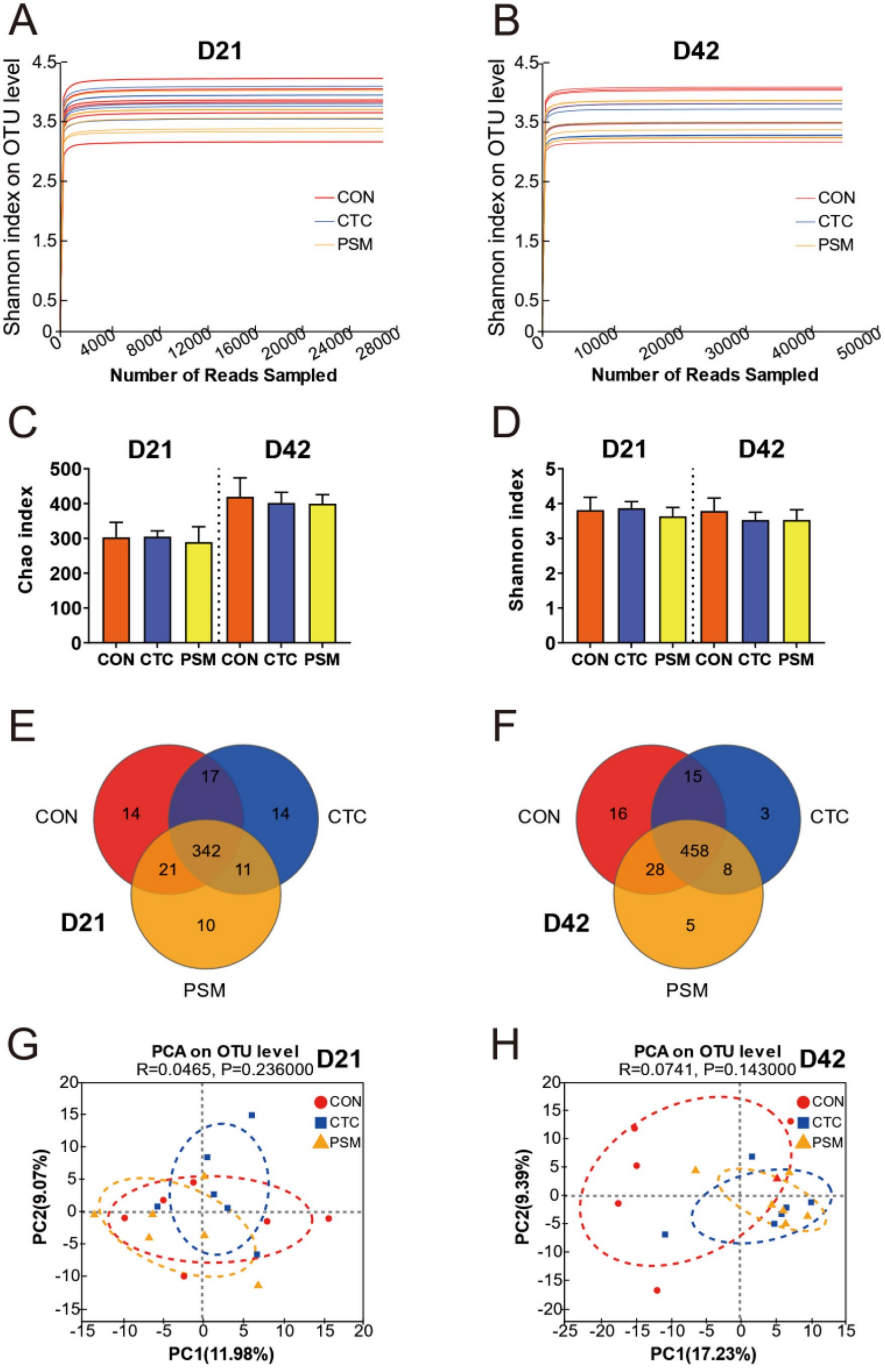
Effects of compound probiotics on microbial community composition. **A**, **B** Rarefaction curves tended to reach the plateau; **C** the alpha-diversity of cecum microbiota in chyme of Chao index; **D** the alpha diversity of cecum microbiota in chyme of Shannon index; **E** VENN diagram at D21; **F** VENN diagram at D42; **G** principal component analysis (PCA) at D21; **H** principal component analysis (PCA) at D42. Data is presented as the mean ± SEM, *n* = 6

To assess the effect of compound probiotics on intestinal microbiota, we profiled the cecal microbial community composition and structure by using 16S rRNA gene sequencing. After applying the previously reported method to remove disqualified sequences, the 18 samples from three groups at D21 were flattened with 27,370 valid sequences and 45,601 valid sequences at D42. The rarefaction curves for all samples approached the plateau indicated that the sequencing depth was sufficient to capture the majority of operational units present in our samples (Fig. 2A and B). Alpha diversity (Chao and Shannon index) revealed the intestinal microbial flora diversity of CTC group and PSM group did not show significantly change compared with CON group (Fig. 2C and D, *P* > 0.05). Furthermore, there were 394, 384, and 384 operational taxonomic units (OTUs) obtained from CON, CTC, and PSM groups at D21, respectively, of which 342 OTUs were common among the three experimental groups. Similarly, 517, 484, and 499 OTUs were classified in CON, CTC, and PSM groups at D42 with 458 common OTUs (Fig. 2E and F). Principal component analysis (PCA) is shown in Fig. 2G and H. At both D21 and D42, PSM group had a strong effect on the beta diversity of the intestinal microbiota in broilers. These results indicated that the compound probiotics supplementation was able to change bacterial community structure in cecum.

**Fig. 3:**
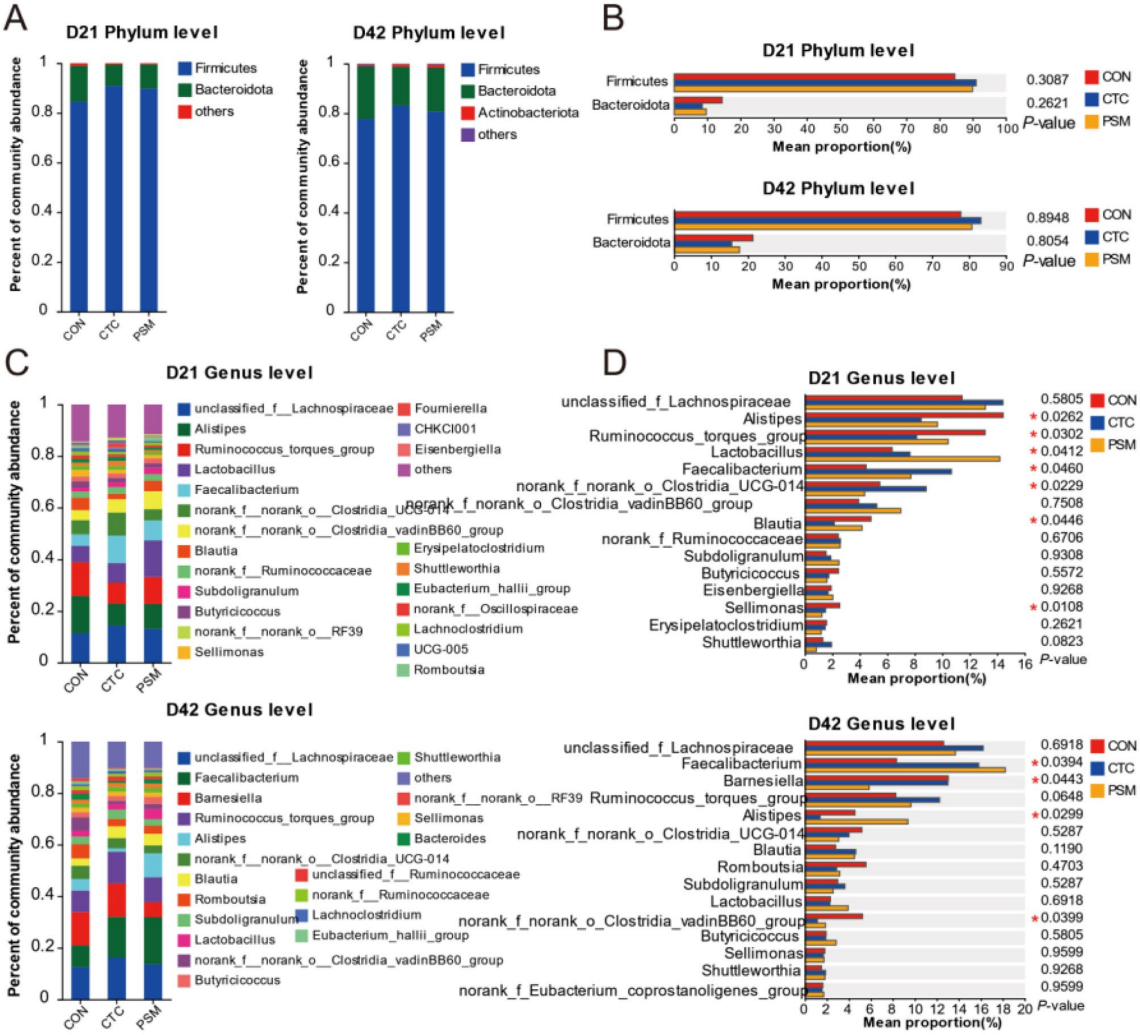
Effects of compound probiotics on the microbiota at the phylum and genus level. **A** The relative abundance of bacterial at the phylum level; **B** the top 2 phylum statistical comparison of the relative abundance in three groups; **C** the relative abundance of bacterial at the genus level; **D** the top 15 genus statistical comparison of the relative abundance in three groups

In terms of the composition of microbial community, *Firmicutes* was the most abundant phylum at both D21- and D42-associated communities in CON, CTC, and PSM groups. Other advantage phyla were *Bacteroidota*, *Proteobacteria*, and *Actinobacteriota* (Fig. 3A). There was no significant difference in the relative abundance of microbiota at the phylum level (Fig. 3B, P > 0.05). Moreover, at the genus level, the composition of the intestinal microbiota included three major genera, *unclassified_f_Lachnospiraceae*, *Alistipes*, and *Ruminococcus_torques_group* at D21 and *unclassified_f_Lachnospiraceae*, *Faecalibacterium*, and *Barnesiella* at D42 (Fig. 3C). In the next step, we compared the top 15 genera in relative abundance among the three groups (Fig. 3D). At D21, the relative abundances of *Lactobacillus* and *Faecalibacterium* were significantly increased in PSM group compared with CON group (*P* < 0.05). However, the relative abundances of *Alistipes* and *Sellimonas* in PSM group were significantly decreased in CON group (*P* < 0.05). The relative abundances of *Faecalibacterium* and *Alistipes* in PSM group were greater than CON group and *Barnesiella* and *norank_f_norank_o_Clostridia_vadinBB60_group* were lower than CON group at D42 (*P* < 0.05). In the case of the CTC group, at D21, *Lactobacillus*, *Faecalibacterium*, and *norank_f_norank_o_Clostridia_UCG-014* have increased, while *Alistipes*, *Ruminococcus_torques_group*, and *Blautia* have reduced compared with CON group (*P* < 0.05). And for D42, only *Faecalibacterium* has increased, while *Alistipes* and *norank_f_norank_o_Clostridia_vandinBB60_group* have decreased (*P* < 0.05). All these findings demonstrated that the addition of chlortetracycline and compound probiotics had no effect on the abundance of intestinal microbiota, but alter their composition in broilers.

### SCFA Concentration in Cecum

**Fig. 4:**
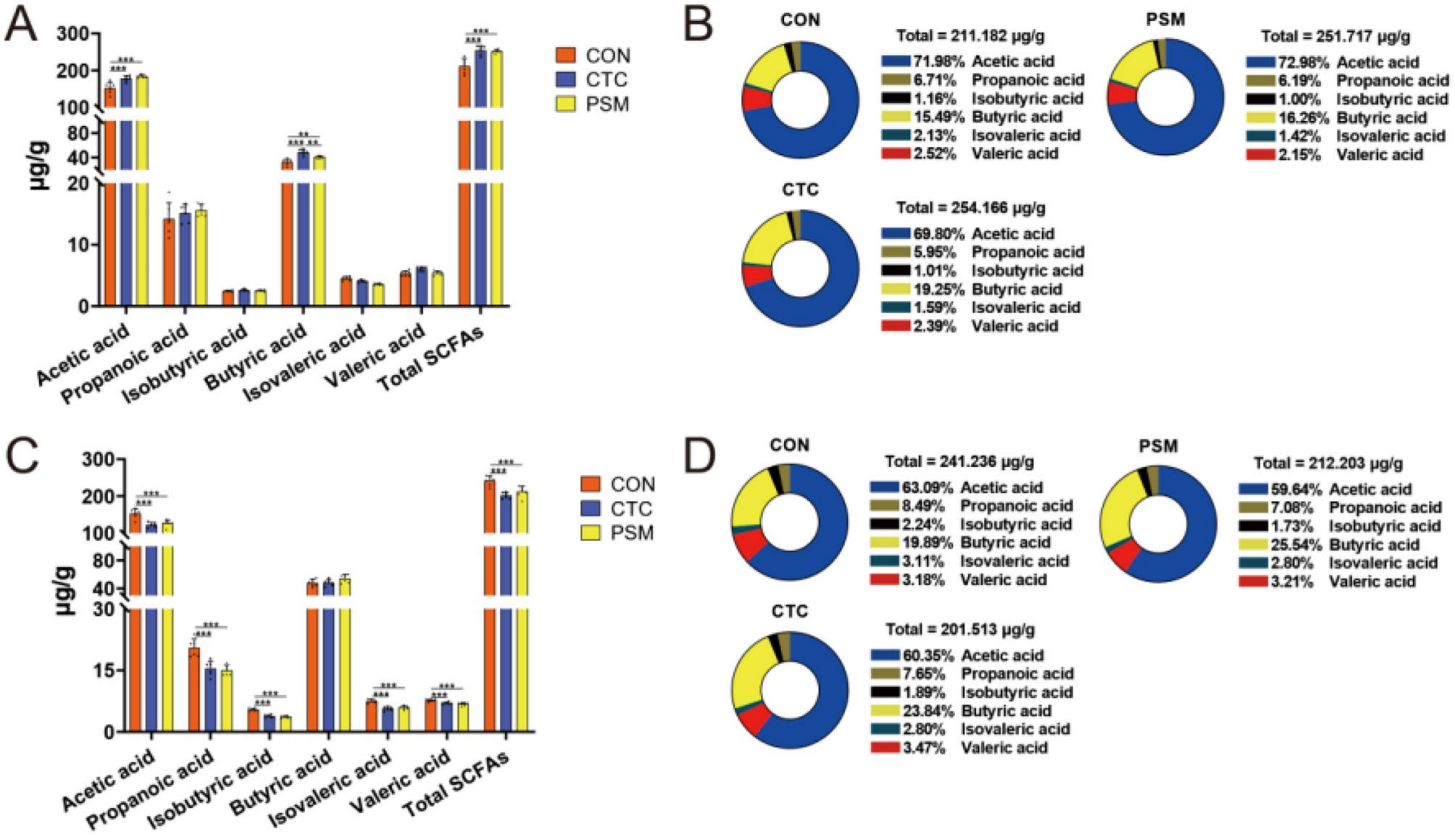
Effects of compound probiotics on SCFAs concentration. **A** SCFA concentration at D21; **B** the proportion of each SCFAs at D21; **C** SCFA concentration at D42; **D** the proportion of each SCFAs at D42 (*n* = 6/group). Data is presented as the mean ± SEM. Signification is presented as *P < 0.05, **P < 0.01, and ***P < 0.001

In order to assess the impact of compound probiotics on the metabolites of the intestinal microbiota, an analysis was conducted on the concentration of short-chain fatty acids (SCFAs) in cecal chyme, as depicted in Fig. 4. At D21, the findings indicated a significant increase in the concentration of acetic acid, butyric acid, and total SCFAs in both CTC group and PSM group when compared to CON group (*P* < 0.01). And there was no significant difference between these three groups in terms of the concentration of propanoic acid, isobutyric acid, isovaleric acid, and valeric acid (*P* > 0.05). While at D42, the results showed that compared with CON group, the concentration of acetic acid, propanoic acid, isobutyric acid, isovaleric acid, valeric acid, and total SCFAs was dramatically reduced in CTC group and PSM group (*P* < 0.01). Butyric acid was the only metabolite that did not significantly change between the three groups (*P* > 0.05). Additionally, the fraction of butyric acid in PSM group was increased despite a reduction in total SCFAs compared with CON group. In general, compound probiotics had beneficial effects on metabolites SCFAs, either by increasing the concentration of total SCFAs or by increasing the concentration of energy-supplying butyric acid.

### The Intestinal Morphology in Duodenum and Jejunum

**Fig. 5:**
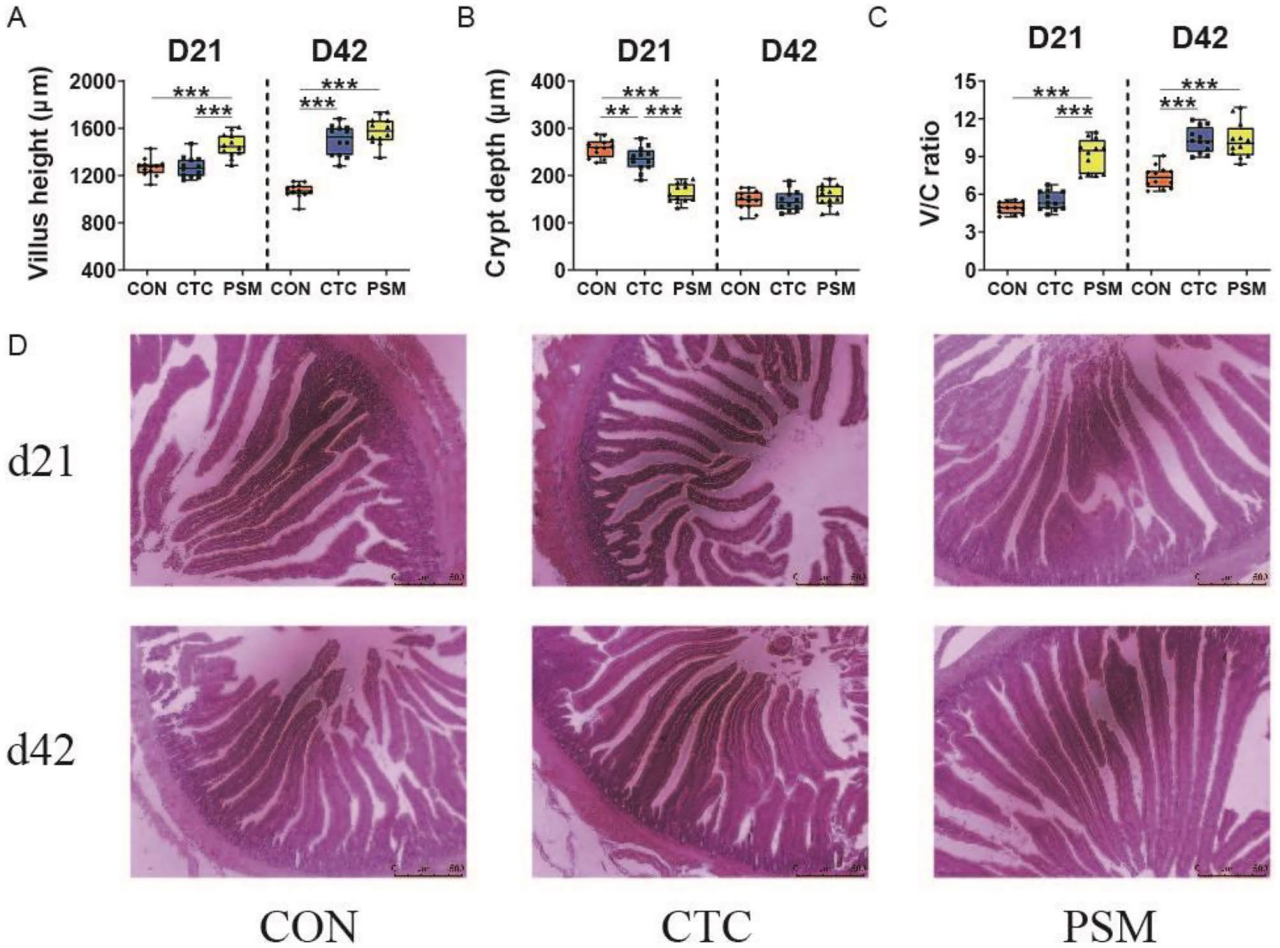
Effects of compound probiotics on intestinal morphology in duodenum of broilers. **A** Villus height in duodenum; **B** crypt depth in duodenum; **C** The ratio of villus height to crypt depth (V/C ratio) in duodenum; **D** The staining profiles by H&E, scale bars 500 μm (*n* = 12/group). Data is presented as the mean ± SEM. Signification is presented as *P < 0.05, **P < 0.01, and ***P < 0.001

**Fig. 6:**
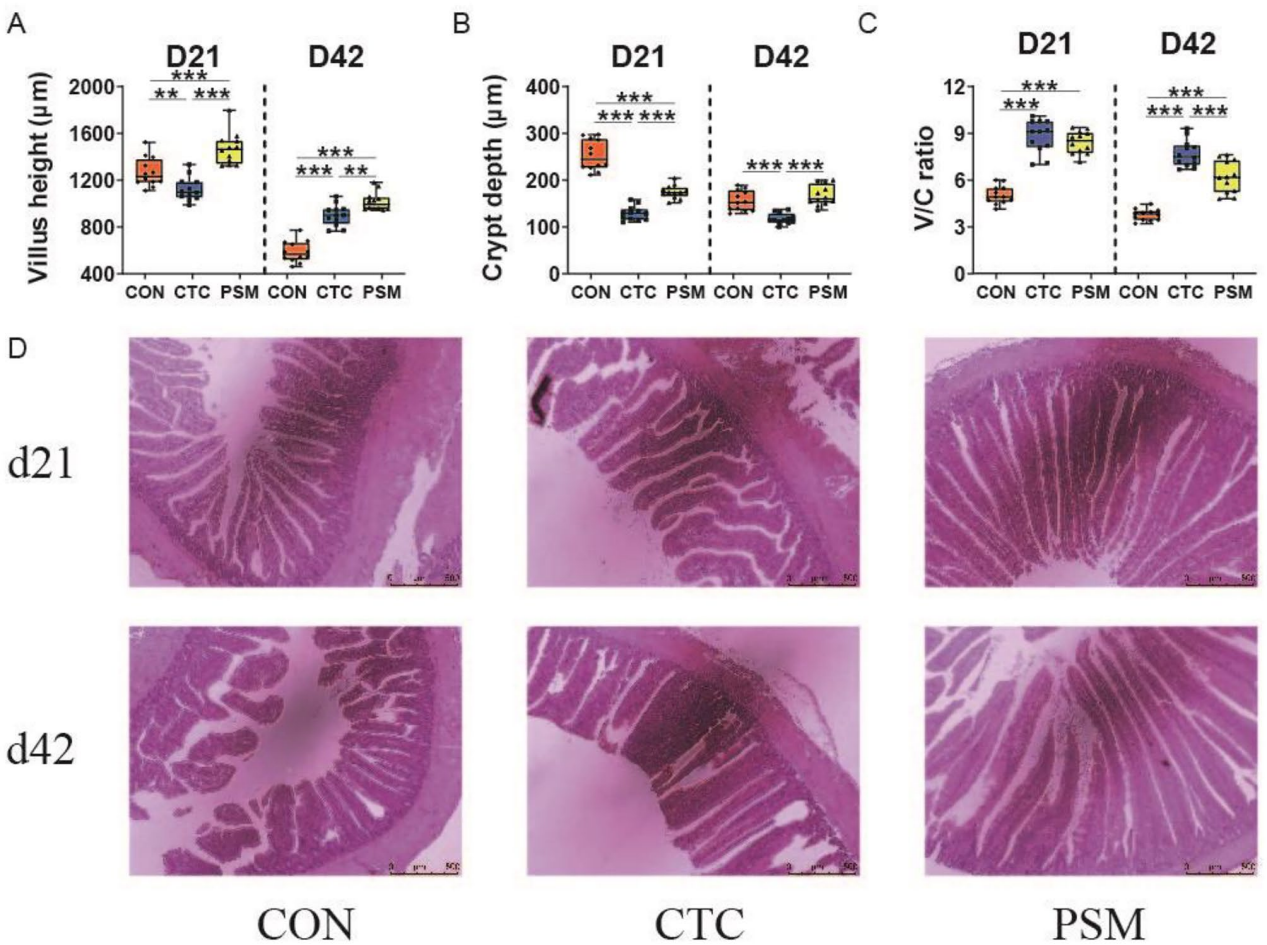
Effects of compound probiotics on intestinal morphology in jejunum of broilers. **A** Villus height in duodenum; **B** crypt depth in duodenum; **C** the ratio of villus height to crypt depth (V/C ratio) in duodenum; **D** The staining profiles by H&E, scale bars 500 μm (n = 12/group). Data is presented as the mean ± SEM. Signification is presented as *P < 0.05, **P < 0.01, and ***P < 0.001

As illustrated by H&E staining of the intestinal morphology and its measurement parameters (Figs. 5 and 6), the duodenum and jejunum of the broilers in CON group were integral, and composed of slender villi and complete crypts. Additionally, the broilers in PSM group also had intact lumens and unbroken villi. Inversely, in CTC group, the villi were tall but sparse, with occurrences of fractures and breakage in the lumen. Besides, the crypts were irregular compared with CON group. In duodenum, villus height and V/C ratio of the broilers in PSM group were increased and crypt depth was reduced compared with CON group and CTC group at D21 (*P* < 0.01). At D42, the villus height and V/C ratio of PSM group were significantly higher than CON group (*P* < 0.01); yet, villus height, crypt depth, and V/C ratio were essentially the same between PSM group and CTC group (*P* > 0.05). In the case of jejunal morphology, broilers in PSM group as well had higher villus height, lower crypt depth, and higher V/C ratio (*P* < 0.01). Nevertheless, the difference is that higher villus, deeper crypt, and decreasing V/C ratio appeared in PSM group compared with CTC group at D42 (*P* < 0.01). The results declared that both chlortetracycline and compound probiotics had positive effects on the intestinal morphology of broilers, and compound probiotics has a stronger positive effect than chlortetracycline.

## Discussion

Probiotics are the most commonly consumed food additives, commonly found in yogurt, cheese, ice cream, snacks, and nutrition bars, and are widely supported by gastroenterologists [27, 28]. Despite the widespread popularity of probiotics, the findings from extensive research conducted over several decades on the effectiveness of probiotics in disease treatment and prevention often yield conflicting conclusions, leading to ongoing debates and discussions [29, 30]. In broiler production, looking through a large number of probiotic use cases, it is found that the addition of probiotics is an important means to improve the production efficiency of broilers and inhibit the occurrence of diseases. Therefore, reasonable and moderate use of appropriate probiotics to guide the production of broilers has a very important industrial significance.

*Enterococcus faecium*, *Bifidobacterium*, and *Pediococcus acidilactici* are considered promising probiotics for maintaining intestinal health and improving production performance, in broiler production [31–33]. It was reported that diet supplement *Enterococcus faecium* remarkably increased ADG (quadratically) and FCR (linearly) in different change forms during the whole feeding process [34]. In addition, body weight gain and FCR of broilers fed *Bifidobacterium* were better than those fed control diets [35]. In terms of *Pediococcus acidilactici*, Wu et al. [36] found that after supplementation, BWG and FI did not increase, but FCR decreased. In this study, the performance of compound probiotics supplementation group was consistent with that of chlortetracycline supplementation group, which was significantly better than the control group. The chlortetracycline and PSM compound probiotics significantly increased BW and ADG and decreased FCR of broilers during the whole production process. However, the mechanism of compound probiotics improving broiler performance is very different from chlortetracycline.

In the present study, feeding with compound probiotics significantly increased the activities of amylase, lipase, trypsin, and chymotrypsin in the duodenum during the whole period. Feeding with compound probiotics had no significant difference in jejunal digestive enzyme activity at D21 compared with the CON group, but significantly increased at D42. Meanwhile, BW and FCR of broilers from PSM group were also improved. These endogenous amylase, lipase, and protease are important for the decomposition, digestion, and absorption of crude proteins, lipids, and carbohydrates from macromolecules to amino acids, triglycerides, and glucose in broilers. Therefore, the increase level of digestive enzyme activities by compound probiotics supplementation could be the reasons for improving the nutrient digestibility and production performance of broilers. It has now been found that the increased activity of digestive enzymes may be caused by the secretion of probiotics digestive enzymes or by the strengthened secretion from cells stimulated by these probiotics, or a combination of these two factors, thereby promoting the secretion of digestive enzymes [37]. Moreover, *Enterococcus faecium* and *Pediococcus acidilactici* could also enhance the development and nutritional function of duodenum and jejunum, which can also encourage the improvement of intestinal enzyme activity [18, 22]. In our study, there was a significant improvement in the intestinal morphological characteristics of broilers after feeding probiotic complexes to enhance the activity of intestinal digestive enzymes. It can be concluded that the activity of intestinal digestive enzymes was affected by exogenous probiotics, and compound probiotics product enhanced the intestinal digestive enzymes activity, which can improve the digestion and absorption rate of broiler chickens, thereby promoting the rapid growth of broilers.

The cecum is the predominant site of fermentation in the digestive tract of broilers and harbors a diverse microbial community. Microorganisms of the cecum profile were directly linked to animal health [38]. These results might be explained by the higher level abundance of *Firmicutes* and lower levels of *Bacteroidetes*, which were previously assumed to be linked to weight gain [39]. The present study reported that broilers fed with compound probiotics increased the ratio of *Firmicutes* to *Bacteroidota* to beneficially modulate the intestinal flora structure. High proportion of *Firmicutes* to *Bacteroidota* of intestinal microbes of broilers was a response to the event of the high-fiber diet resources, and it could help broilers to obtain more energy from dietary food [40]. In addition, the difference in nutrient absorption caused by intestinal permeability and intestinal wall thickness, the influence of microorganisms on material metabolism, and their own metabolites may also affect body weight. Many previous studies confirmed that *Enterococcus faecium*, *Bifidobacterium*, and *Pediococcus acidilactici* can improve microbial composition, microbial metabolites, and intestinal morphometry [18, 19, 21]. Meanwhile, some studies found that the addition of probiotics had neutral or negative effects on growth performance and intestinal microorganisms of broilers, like that the addition of *B. subtilis* and *E. faecium* will destroy the original microbial structural balance and lead to a decrease in the average weight gain of broilers [41]. The reason for this result may be that different types of microorganisms have different effects on intestinal health or that microorganisms consume too much energy from diet, which brings negative effects on production performance. In conclusion, the compound probiotics selected in this experiment are effective on the intestinal microbial balance of broilers and can be further applied in production practice to replace antibiotics to achieve efficient production and animal health.

In recent years, as an important derived metabolite of the intestinal microflora, SCFAs have drawn greater attention [42]. SCFAs can promote intestinal health, especially, acetic acid, propanoic acid, and butyric acid can be directly absorbed as a nutrient and help maintain intestinal mucosal integrity, they became one of the most noteworthy SCFAs [43, 44]. In this study, we investigated intestinal microbial metabolites and found that there was an improvement in the concentration of acetic acid, butyric acid and total SCFAs of broilers fed compound probiotics supplementation diets at D21. These results were attributed to the increased abundance of SCFA producing bacteria, which is critical for SCFA production, by the supplementation of compound probiotics. Among the top 15 genera, *Ruminococcus_torques_group*, *Lactobacillus*, *Faecalibacterium*, *norank_f_norank_o_Clostrida_UCG-014*, *norank_f_norank_o_Clostridia_vadinBB60_group*, and *Butyricicoccus* have been documented as a bacterium that exerts SCFA-producing capabilities [45–47]. In agreement with our studies, previous studies reported that broilers fed probiotics supplementation diets had higher SCFA production at D21 cecum compared with broilers fed diet without probiotics supplementation [48]. In this study, the supplementation of compound probiotics increased the abundance of SCFA-producers such as *Lactobacillus*, *Faecalibacterium*, and *norank_f_norank_o_Clostridia_vadinBB60_group*, consequently resulting in an increment in SCFA levels. Moreover, intestinal microbiota primarily produces SCFAs by fermenting monosaccharides derived from nutrients and mucins, with acetate and butyrate being promoted mainly by bacteria such as *Ruminococcus* spp. and *Lactobacillus* spp. [49]. This also explains the higher concentrations of cecal acetate and total SCFA in compound probiotics supplementation. Furthermore, some studies have revealed that SCFAs can increase intestinal acidity, such as butyrate and propionate, which have inhibitory effects on foodborne pathogen. However, the results of studies on SCFA levels in cecal digest of broilers at different days age are inconsistent. Czerwiński et al. [50] added probiotics to broiler diets and found that the concentrations of acetic acid, butyric acid as well as total SCFAs in the intestine were significantly reduced following addition of the dietary probiotic. Similarly, within this study, while an increment in SCFA-producers was observed at D42, there was a concurrent decline in the concentration of total SCFAs in the PSM group. Since the concentration of SCFAs in cecum is affected by many factors [51], the physiological role of SCFAs in different growth stages needs to be further studied.

Intestinal morphology is the most intuitive manifestation of the health and integrity of the digestive tract, including villus height, crypt depth, and V/C ratio. Increased villus height indicates a larger area for nutrient absorption in the intestine and more mature enterocytes accumulating, which representing enhanced absorptive capacity [52]. Shorter villi and deeper crypts were observed, suggesting reduced digestive and absorptive function. The ratio of V/C can reflect the turnover rate of intestinal cells [53]. Consistent with our findings, numerous studies have shown that dietary supplementation with probiotics can affect intestinal morphological parameters [54]. In this study, we demonstrated that the PSM group showed significantly increased villus height and V/C ratio, and decrease crypt depth in ileum at D21. This findings were in agreement with the results of Xie et al. [54], who showed that the addition of probiotics improved the intestinal morphology of the experimental chickens, including increased villus height and V/C ratio, and decreased crypt depth. Huang et al. [18] also found that the addition of *Faecalibacterium* improved intestinal histomorphology, increasing the V/C ratio and villus height in infected birds. Improvements in intestinal morphology may be indirectly due to improved nutritional status (SCFAs, amino acids, and others) causing fermentation of metabolites [55]. The administration of compound probiotics has been shown to enhance the abundance of beneficial bacteria, including *Lactobacillus*, *Faecalibacterium*, and *norank_f_norank_o_Clostridia_vadinBB60_group*, thereby facilitating improved nutrient digestion and absorption. Simultaneously, compound probiotics increased the production of SCFAs, particularly propionic acid and butyric acid, which were conducive to intestinal growth and development, enhancing intestinal health and feed conversion efficiency in broiler chickens. Such results revealed that compound probiotics had an improved effect on intestinal morphology, which may help to promote the absorption of nutrients in broilers, thereby enhancing growth performance.

As normal inhabitants of diverse ecosystems, including the gastrointestinal tract, *Enterococcus faecium*, *Bifidobacterium*, and *Pediococcus acidilactici* can be considered critical to intestinal microecology. *Enterococcus faecium* does not colonize permanently in animals; often, temporary colonization occurs after administration with the decreasing number of *Enterococcus faecium* CFU over time [56]. Therefore, this study selected the supplementation of compound probiotics for the whole production of broilers and found that after the supplementation, the activity of digestive enzymes in broilers was significantly increased and generation direction of SCFAs, microbiota structure, and intestinal morphology was significantly improved. Research into the beneficial properties of compound probiotics has unveiled a range of mechanisms, including the production of bioactive molecules, such as SCFAs, and digestive enzymes. One of the reasons for the physiological effect of compound probiotics on the growth performance of broilers is to improve the activity of a series of digestive enzymes such as amylase, lipase, trypsin and chymotrypsin, which is a direct reflection of the improvement of intestinal nutrient metabolism ability of broilers. Acetic acid can be used by butyric acid producing bacteria in the intestine, such as *Faecalibacterium*, to produce butyric acid [57]. Butyric acid is used by intestinal epithelial cells as an energy source and is involved in a variety of physiological functions, including intestinal barrier function and immune function [58, 59]. In the intestine, *Bifidobacterium* metabolizes carbohydrates to SCFAs, acetate, and lactate [60]. Although bifidobacteria do not directly synthesize butyric acid, their production of acetic acid may influence the activity and composition of other members of the gut microbiota that produce butyric acid, thereby stimulating the secondary butyric acid effect [61, 62]. In addition, this study also found that the supplementation of compound probiotics directly increased the relative abundance of intestinal *Faecalibacterium* and drive SCFA optimization in the direction of butyric acid generation, which further explained the reasons for the improvement of intestinal morphology and the improvement of feed efficiency. Although the compound probiotics in this study had good effects on growth performance, intestinal microbiota, and intestinal tissue morphology of broilers, and were consistent with the results of current high-quality studies, there were still contrary conclusions, and the differences may be attributed to the experimental design of different studies, different measurement parameters, and experimental conditions. Probiotics secrete many substances (metabolites), and there are many possible pathways to action, most of which have not been fully elucidated [63]. The complex ratio of compound probiotics and the difference in the proportion of active ingredients are the important reasons for the differences in experimental results. Secondly, “strain difference” — in recent years, human microecological studies have emphasized that the research needs to be refined to the “strain level.” The probiotic species and strains used in experimental research are different, and the combination of multiple strains makes it more difficult to elucidate the health effects of probiotics [29]. In addition, the standardization of animal models and production of probiotics also affect the research and clinical application of probiotics. In the future, more production trials of compound probiotics and more optimization and standardization of the ratio will help provide a more powerful basis for more widespread application in the use of antibiotic replacement.

## Conclusion

In summary, supplementation of compound probiotics based on *Enterococcus faecium*, *Bifidobacterium*, and *Pediococcus acidilactici* in diets enhanced growth performance of broiler chickens, manifested by increasing BW, ADG, and reducing FCR. During this process, compound probiotics enhanced the relative abundance of beneficial bacteria *Lactobacillus*, *Faecalibacterium*, and *norank_f_norank_o_Clostridia_vadinBB60_group*, thereby optimizing the intestinal microbiota. The increase in SCFAs produced by probiotics is beneficial to increase villus height, decrease crypt depth, and increase villus/crypt ratio. In addition, the stimulation of the well-developed intestine by probiotics promotes the enhancement of intestinal digestive enzyme activity. Therefore, the use of compound probiotics improved the growth performance of broilers by regulating the intestinal microbial community to promote early intestinal development.

## Data Availability

All 16S rRNA Illumina amplicon sequencing data provided in this study can be publicly obtained in the Sequence Read Archive (SRA) of the National Center for Biotechnology Information (NCBI) under the accession number SRP PRJNA947554. The datasets generated during and analyzed during the current study are available from the corresponding author on reasonable request.
